# Supplementary material for: Genotypic variation in disease susceptibility among cultured stocks of elkhorn and staghorn corals
Source: PeerJ. 2019 Apr 8;7:e6751. doi: 10.7717/peerj.6751 (PMC6459175; doi:10.7717/peerj.6751)
Supplement: Supplemental Information 1 — (A–B) “healthy” allograft control after 7 days illustrating slight abrasion of raised tissue, but no tissue-loss lesions as observed in disease transmission (C) day 0, (D) Day 3, (E) close-up of elicited lesion). [file peerj-07-6751-s001.pdf]

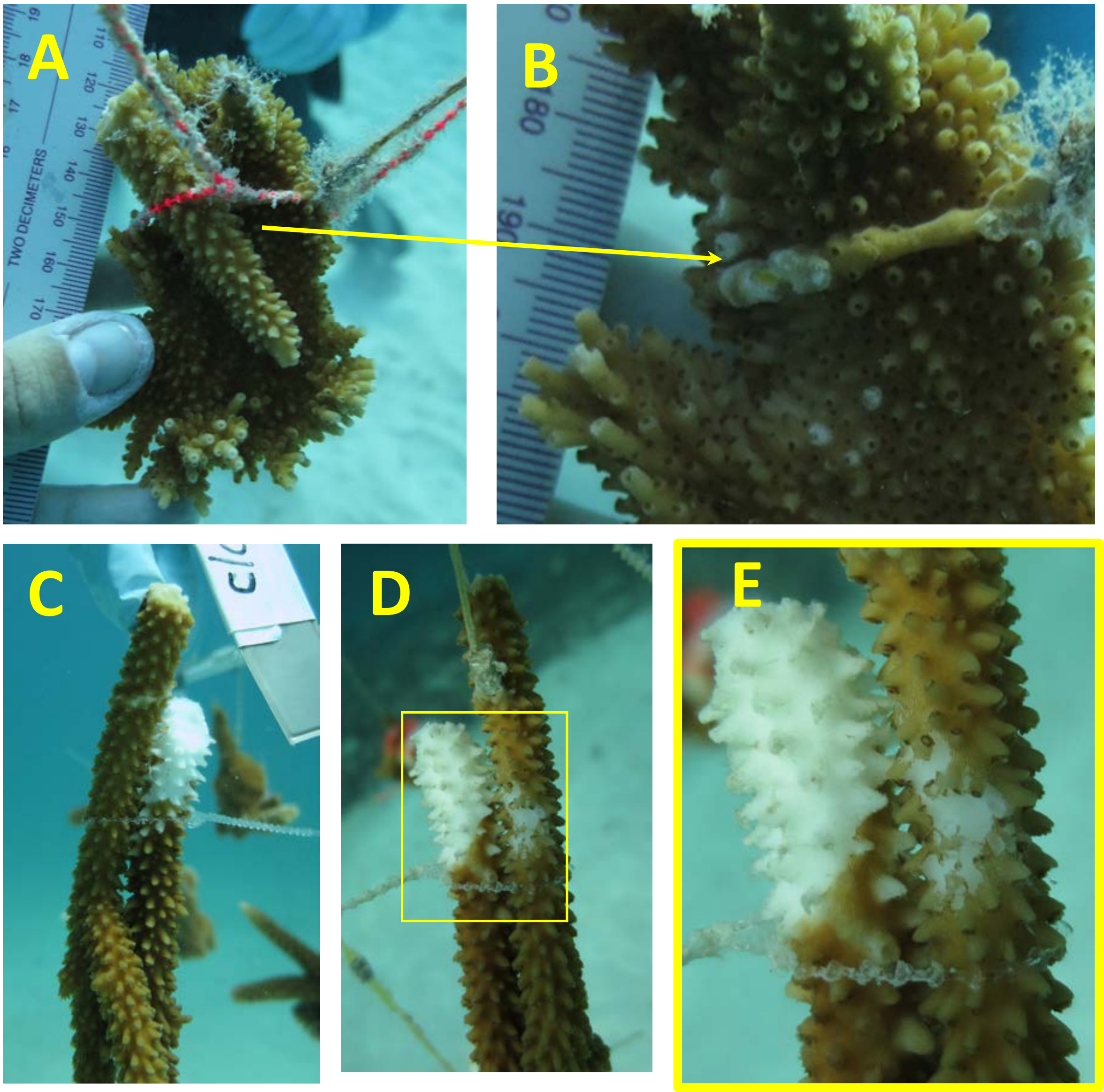

Suppl Fig 1: A-B)‘healthy’ allograft control after 7 days illustrating slight abrasion of raised tissue, but no tissue-loss lesions as observed in disease transmission (C: day 0, D: Day 3, E:close-up of elicited lesion)
