# Supplementary material for: Genotypic variation in disease susceptibility among cultured stocks of elkhorn and staghorn corals
Source: PeerJ. 2019 Apr 8;7:e6751. doi: 10.7717/peerj.6751 (PMC6459175; doi:10.7717/peerj.6751)
Supplement: Supplemental Information 2 — Photomicrographs of results from the histopathological examinations of 2016 and 2017 samples in this study (A–G) revealing (A) histophagous Symbiodinium (S)-containing ciliates (CI) adjacent to pieces of degraded epidermis (e), note complete loss of gastrodermis, H&E, scale bar = 100 µm; (B) suspect Rickettsiales-like organisms (RLOs) stain dark purple in mucocytes of cnidoglandular bands of mesenterial filaments, Giemsa, scale bar = 200 µm; (C) thick layer of mucus produced by hypertrophied surface body wall mucocytes trapped under agarose in a rapid tissue loss-affected sample from 2017, H&E, scale bar = 200 µm; (D) suspect bacteria (b) staining dark blue on paler blue mucus strands from a 2017 inoculant A. cervicornis sample, Giemsa, scale bar = 100 µm; (E) apoptosis of calicoblast (black circle) in calicodermis (c) of basal body wall at tissue-loss margin, note that this area of calicodermis (at arrow) contains hypertrophied calicoblasts producing numerous fine granules of coral acid-rich proteins (CARPs) compared to the epithelium lining the other side of skeleton (SK; removed by decalcification procedure) composed of squamous calicoblasts, indicating more active skeletal production or wound repair, and the calicodermis and gastrodermis (g) containing Symbiodinium (S) are also vacuolated, H&E, scale bar = 50 µm; (F) section through another area of abnormal basal body wall at tissue-loss margin, calicodermis (c) composed of hypertrophied columnar cells, adjacent gastrodermis (g) also has hypertrophied cells compared to the gastrodermis lining the squamous calicodermis where the skeleton (SK) was removed, acute degradation of epithelia by necrosis or apoptosis (N-A), H&E, scale bar = 100 µm; (G) unusually large example of formation of ball of degraded cells and debris (N) surrounded by what appears to be a layer of live calicoblasts (c) that form at the tissue-loss margin and can be trapped by the agarose, this one containing degraded ova (O) and mesenteries with [file peerj-07-6751-s002.pdf]

Supplemental Figure 2: Photomicrographs of results from the histopathological examinations of 2016 and 2017 samples in this study (A–G) revealing A) histophagous *Symbiodinium* (S)-containing ciliates (CI) adjacent to pieces of degraded epidermis (e), note complete loss of gastrodermis, H&E, scale bar = 100 µm; B) suspect Rickettsiales-like organisms (RLOs) stain dark purple in mucocytes of cnidoglandular bands of mesenterial filaments, Giemsa, scale bar = 200 µm; C) thick layer of mucus produced by hypertrophied surface body wall mucocytes trapped under agarose in a rapid tissue loss-affected sample from 2017, H&E, scale bar = 200 µm; D) suspect bacteria (b) staining dark blue on paler blue mucus strands from a 2017 inoculant *A. cervicornis* sample, Giemsa, scale bar = 100 µm; E) apoptosis of calicoblast (black circle) in calicodermis (c) of basal body wall at tissue-loss margin, note that this area of calicodermis (at arrow) contains hypertrophied calicoblasts producing numerous fine granules of coral acid-rich proteins (CARPs) compared to the epithelium lining the other side of skeleton (SK; removed by decalcification procedure) composed of squamous calicoblasts, indicating more active skeletal production or wound repair, and the calicodermis and gastrodermis (g) containing *Symbiodinium* (S) are also vacuolated, H&E, scale bar = 50 µm; F) section through another area of abnormal basal body wall at tissue-loss margin, calicodermis (c) composed of hypertrophied columnar cells, adjacent gastrodermis (g) also has hypertrophied cells compared to the gastrodermis lining the squamous calicodermis where the skeleton (SK) was removed, acute degradation of epithelia by necrosis or apoptosis (N-A), H&E, scale bar = 100 µm; G) unusually large example of formation of ball of degraded cells and debris (N) surrounded by what appears to be a layer of live calicoblasts (c) that form at the tissue-loss margin and can be trapped by the agarose, this one containing degraded ova (O) and mesenteries with terminal mesenterial filament cnidoglandular bands (CGB) H&E, scale bar = 500 µm; H) section of tentacle epidermis with loss of mucocytes (M) and abundant turquoise-staining specks in the gastrodermal cells (g), the densest aggregations of these specks lack intact algal cells (S).

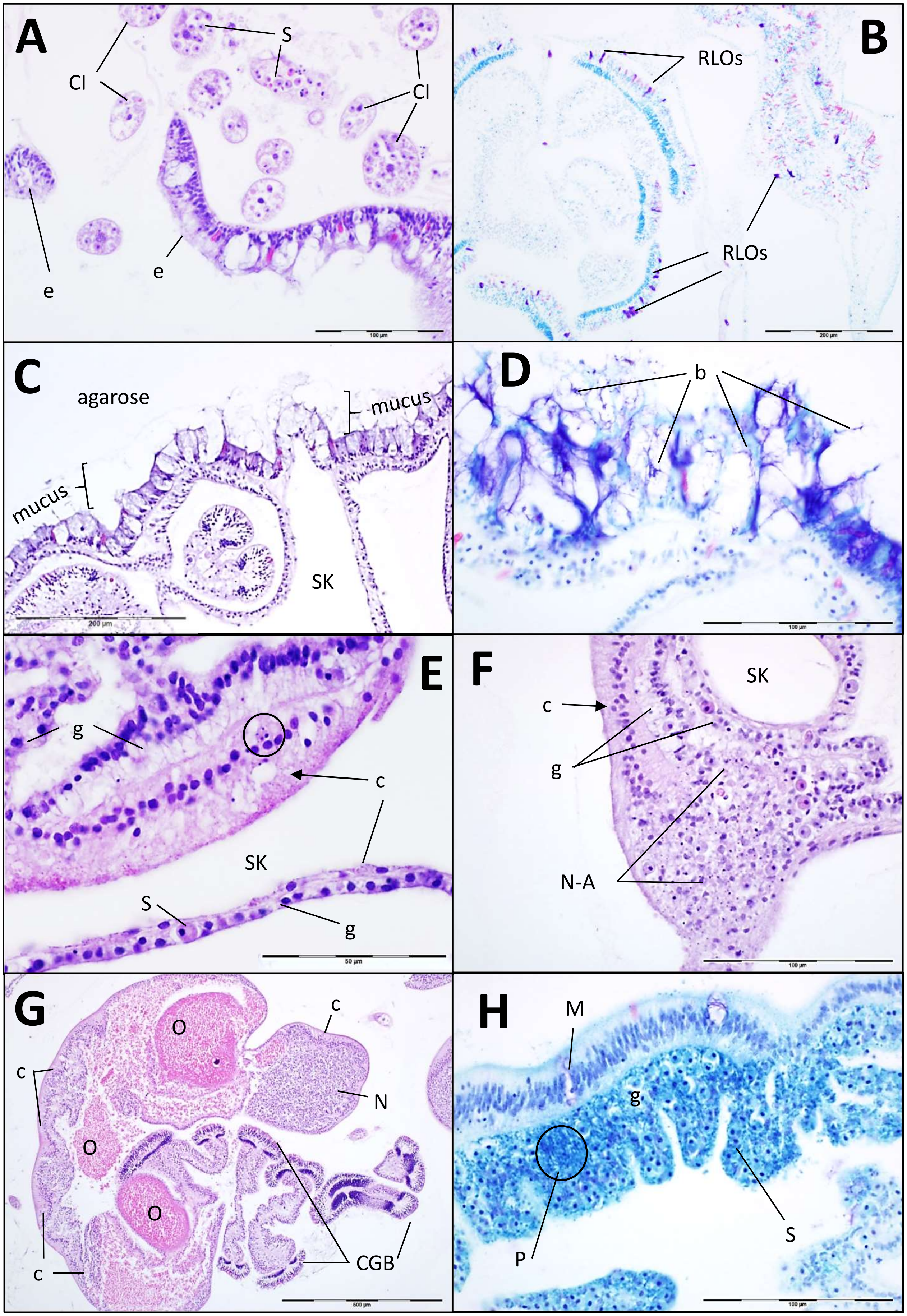
A
S
CI
CI
e
e
100 µm
B
RLOs
RLOs
200 µm
C
agarose
mucus
mucus
SK
200 µm
D
b
100 µm
E
g
c
c
SK
S
g
50 µm
F
SK
c
g
N-A
100 µm
G
O
c
c
N
O
O
c
c
CGB
500 µm
H
M
g
P
S
100 µm
