# Supplementary material for: Genotypic variation in disease susceptibility among cultured stocks of elkhorn and staghorn corals
Source: PeerJ. 2019 Apr 8;7:e6751. doi: 10.7717/peerj.6751 (PMC6459175; doi:10.7717/peerj.6751)
Supplement: Supplemental Information 3 — Supplemental Figure 3: FISH for suspect bacteria using eubacterial probes (A–D) revealing (A) small coccoid bacteria (b) labeled with EUB338-I probe in coenenchyme surface body wall epidermis from 2016 WBD-affected A. cervicornis inoculant sample (16-177-2-2-1), red autofluorescing Symbiodinium (S) in gastrodermis, scale bar = 50 µm; (B) more abundant minute coccoid bacteria (b) labeled with EUB338-I probe in coenenchyme surface body wall epidermis of 2017 RTL-affected A. cervicornis inoculant sample (17-054-4-2-1), note more abundant and hypertrophied mucocytes, Symbiodinium (S) seen by their red autofluorescence, scale bar = 20 µm; (C) fewer bacteria (b) in epidermis, some in gastrodermis from same sample as A, 2016 A. cervicornis WBD-affected inoculant labeled using EUB338-II probe for Planctomycetales bacteria, red autofluorescing Symbiodinium (S), scale bar = 50 µm; (C) more abundant bacteria in epidermis of same 2017 A. cervicornis inoculant sample surface body wall labeled by EUB338-II probe, paler Symbiodinium (S) and few bacteria in gastrodermis, scale bar = 50 µm; (E) minute coccoid bacteria (b) with EUB338-I probe primarily on surface of degraded cells along tissue-loss margin in 2016 WBD-affected A. cervicornis inoculant sample (16-177-2-2-1), Symbiodinium (S) seen by their red autofluorescence, scale bar = 20 µm; (b) more coccoid bacteria (F) in the same locations on a serial section from the same paraffin block shown using EUB338-II probe for Planctomycetales bacteria, red autofluorescing Symbiodinium (S), scale bar = 20 µm. [file peerj-07-6751-s003.pdf]

Supplemental Figure 3: FISH for suspect bacteria using eubacterial probes (A–D) revealing A) small coccoid bacteria (b) labeled with EUB338-I probe in coenenchyme surface body wall epidermis from 2016 WBD-affected *A. cervicornis* inoculant sample (16-177-2-2-1), red autofluorescing *Symbiodinium* (S) in gastrodermis, scale bar = 50 µm; b) more abundant minute coccoid bacteria (b) labeled with EUB338-I probe in coenenchyme surface body wall epidermis of 2017 RTL-affected *A. cervicornis* inoculant sample (17-054-4-2-1), note more abundant and hypertrophied mucocytes, *Symbiodinium* (S) seen by their red autofluorescence, scale bar = 20 µm; C) fewer bacteria (b) in epidermis, some in gastrodermis from same sample as A, 2016 *A. cervicornis* WBD-affected inoculant labeled using EUB338-II probe for Planctomycetales bacteria, red autofluorescing *Symbiodinium* (S), scale bar = 50 µm; C) more abundant bacteria in epidermis of same 2017 *A. cervicornis* inoculant sample surface body wall labeled by EUB338-II probe, paler *Symbiodinium* (S) and few bacteria in gastrodermis, scale bar = 50 µm; E) minute coccoid bacteria (b) with EUB338-I probe primarily on surface of degraded cells along tissue-loss margin in 2016 WBD-affected *A. cervicornis* inoculant sample (16-177-2-2-1), *Symbiodinium* (S) seen by their red autofluorescence, scale bar = 20 µm; b) more coccoid bacteria (F) in the same locations on a serial section from the same paraffin block shown using EUB338-II probe for Planctomycetales bacteria, red autofluorescing *Symbiodinium* (S), scale bar = 20 µm.

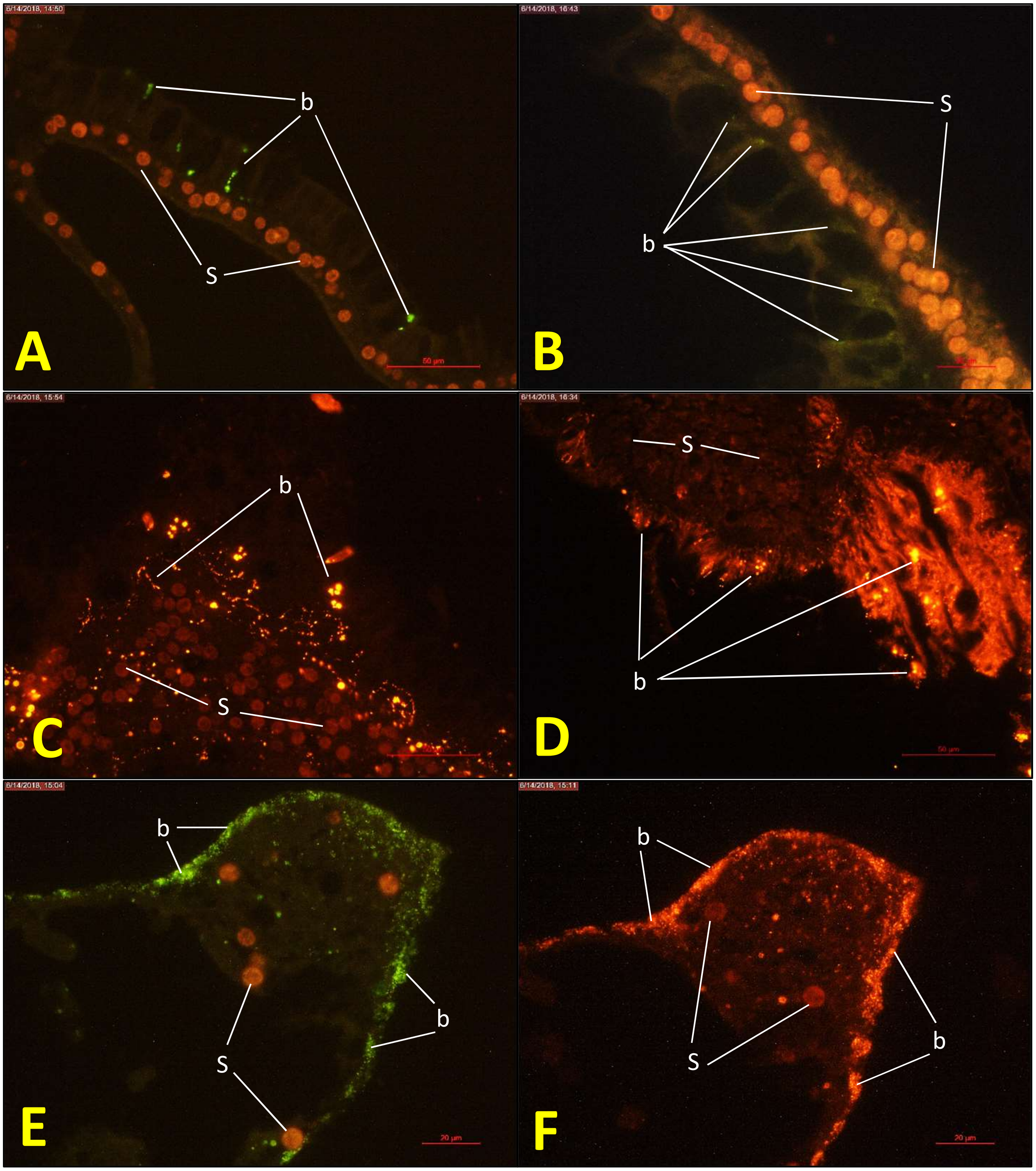
6/14/2018, 14:50
b
S
50 µm
A
6/14/2018, 16:43
S
b
B
6/14/2018, 15:54
b
S
C
6/14/2018, 16:34
S
b
50 µm
D
6/14/2018, 15:04
b
b
S
20 µm
E
6/14/2018, 15:11
b
b
S
20 µm
F
