# Supplementary material for: Genotypic variation in disease susceptibility among cultured stocks of elkhorn and staghorn corals
Source: PeerJ. 2019 Apr 8;7:e6751. doi: 10.7717/peerj.6751 (PMC6459175; doi:10.7717/peerj.6751)
Supplement: Supplemental Information 4 — Summary of Acropora cervicornis genotypes screened during the 2-year study. CRF, Coral Restoration Foundation, Tavernier; FWC, Florida Fish and Wildlife Conservation Commission, Marathon; UM, Univ of Miami, Key Biscayne. Msat, genotypes determined by microsatellite markers (Baums et al., 2009), GBS, Genotyping by Sequencing (Drury et al., 2016) , NA indicates no specific analyses have been done to verify so genotype identity is putative. N = number of replicate fragments of each genotype represented in the susceptibility transmission risk (Figs. 1A and 1B). [file peerj-07-6751-s004.docx]

Suppl Table 1: Summary of *Acropora cervicornis* genotypes screened during the 2-year study. CRF = Coral Restoration Foundation, Tavernier; FWC= Florida Fish and Wildlife Conservation Commission, Marathon; UM = Univ of Miami, Key Biscayne. Msat = genotypes determined by microsatellite markers (Baums et al., 2009), GBS = Genotyping by Sequencing (Drury et al., 2016), NA indicates no specific analyses have been done to verify so genotype identity is putative. N = number of replicate fragments of each genotype represented in the susceptibility transmission risk (Fig 1A&B).

| Year | Genotype designation in this study (see Fig 1) | Nursery of origin | Genotype name | Genotyping method | N |
| --- | --- | --- | --- | --- | --- |
| 2016 | C1 | CRF | U41 | Msat | 9 |
| 2016 | C2 | CRF | M5 | Msat | 8 |
| 2016 | C3 | CRF | U44 | Msat | 8 |
| 2016 | C4 | CRF | U77 | Msat | 8 |
| 2016 | C5 | CRF | U25 | Msat | 9 |
| 2016 | C6 | CRF | K2 | Msat | 9 |
| 2016 | C7 | FWC | 20 | Msat | 8 |
| 2016 | C8 | FWC | 6 | Msat | 8 |
| 2016 | C9 | FWC | 9 | Msat | 9 |
| 2016 | C10 | FWC | 13 | Msat | 8 |
| 2016 | C11 | FWC | M6 | Msat | 8 |
| 2016 | C12 | FWC | 3 | Msat | 8 |
| 2016 | C13 | FWC | 19 | Msat | 8 |
| 2016 | C14 | FWC | 18 | Msat | 8 |
| 2016 | C15 | FWC | M5 | Msat | 8 |
| 2016 | C16 | FWC | 14 | Msat | 8 |
| 2017 | C17 | UM | Kelsey | NA | 10 |
| 2017 | C18 | UM | KBCF-32 | NA | 10 |
| 2017 | C19 | UM | Sunny-33 | GBS | 10 |
| 2017 | C20 | UM | Stag | NA | 10 |
| 2017 | C21 | UM | Elkhorn | Msat, GBS | 10 |
| 2017 | C22 | UM | POM3 | GBS | 10 |
| 2017 | C23 | UM | MB1 | NA | 10 |
| 2017 | C24 | UM | Cooper-9 | Msat, GBS | 10 |
| 2017 | C25 | UM | MB2 | NA | 10 |
| 2017 | C26 | UM | Acerv2 | MSat, GBS | 10 |
| 2017 | C28 | FWC | C1398 | Msat | 10 |
| 2017 | C29 | FWC | 21 | Msat | 10 |
| 2017 | C30 | FWC | 23 | Msat | 10 |
| 2017 | C31 | FWC | 25 | Msat | 10 |
