# Supplementary material for: Genotypic variation in disease susceptibility among cultured stocks of elkhorn and staghorn corals
Source: PeerJ. 2019 Apr 8;7:e6751. doi: 10.7717/peerj.6751 (PMC6459175; doi:10.7717/peerj.6751)
Supplement: Supplemental Information 5 — CRF, Coral Restoration Foundation, Tavernier; Msat, microsatellite markers (Baums et al., 2003, 2009), MtDNA indicates mitrochondrial control region (Vollmer & Palumbi, 2007; Van Oppen et al., 1999) , N indicates the number of replicate fragments of each genotype represented in the susceptibility transmission risk (Figs. 1A and 1B). [file peerj-07-6751-s005.docx]

Suppl Table 2: Summary of *Acropora palmata* genotypes screened during the 2-year study. CRF = Coral Restoration Foundation, Tavernier; Msat= microsatellite markers (Baums et al. 2003, 2009), MtDNA indicates mitrochondrial control region (Vollmer and Palumbi, 2007, van Oppen et al., 1999), N indicates the number of replicate fragments of each genotype represented in the susceptibility transmission risk (Fig 1A&B).

| Year | Genotype designation in this study (see Fig 1) | Nursery of origin | Genotype name | Genotyping method | N |
| --- | --- | --- | --- | --- | --- |
| 2016 | P1 | CRF | HS1 | Msat | 9 |
| 2016 | P2 | CRF | CN1 | mtDNA | 9 |
| 2016 | P3 | CRF | ML6 | mtDNA | 7 |
| 2016 | P4 | CRF | CN2 | mtDNA | 10 |
| 2016 | P5 | CRF | SL | Msat | 8 |
| 2016 | P6 | CRF | CN3 | mtDNA | 10 |
| 2017 | P7 | CRF | CN4 | mtDNA | 10 |
| 2017 | P8 | CRF | ML2 | mtDNA | 10 |
| 2017 | P9 | CRF | SI5 | Msat | 10 |
| 2017 | P10 | CRF | SI1 | Msat | 10 |
| 2017 | P11 | CRF | AAA3 | mtDNA | 10 |
| 2017 | P12 | CRF | AAA2 | mtDNA | 10 |
